# Penicillin Induced Persistence in *Chlamydia trachomatis*: High Quality Time Lapse Video Analysis of the Developmental Cycle

**DOI:** 10.1371/journal.pone.0007723

**Published:** 2009-11-06

**Authors:** Rachel J. Skilton, Lesley T. Cutcliffe, David Barlow, Yibing Wang, Omar Salim, Paul R. Lambden, Ian N. Clarke

**Affiliations:** Molecular Microbiology Group, University of Southampton Medical School, Southampton General Hospital, Southampton, United Kingdom; University of California Los Angeles, United States of America

## Abstract

**Background:**

*Chlamydia trachomatis* is a major human pathogen with a unique obligate intracellular developmental cycle that takes place inside a modified cytoplasmic structure known as an inclusion. Following entry into a cell, the infectious elementary body (EB) differentiates into a non - infectious replicative form known as a reticulate body (RB). RBs divide by binary fission and at the end of the cycle they redifferentiate into EBs. Treatment of *C.trachomatis* with penicillin prevents maturation of RBs which survive and enlarge to become aberrant RBs within the inclusion in a non - infective persistent state. Persistently infected individuals may be a reservoir for chlamydial infection. The *C.trachomatis* genome encodes the enzymes for peptidoglycan (PG) biosynthesis but a PG sacculus has never been detected. This coupled to the action of penicillin is known as the chlamydial anomaly. We have applied video microscopy and quantitative DNA assays to the chlamydial developmental cycle to assess the effects of penicillin treatment and establish a framework for investigating penicillin induced chlamydial persistence.

**Principal Findings:**

Addition of penicillin at the time of cell infection does not prevent uptake and the establishment of an inclusion. EB to RB transition occurs but bacterial cytokinesis is arrested by the second binary fission. RBs continue to enlarge but not divide in the presence of penicillin. The normal developmental cycle can be recovered by the removal of penicillin although the large, aberrant RBs do not revert to the normal smaller size but remain present to the completion of the developmental cycle. Chromosomal and plasmid DNA replication is unaffected by the addition of penicillin but the arrest of bacterial cytokinesis under these conditions results in RBs accumulating multiple copies of the genome.

**Conclusions:**

We have applied video time lapse microscopy to the study of the chlamydial developmental cycle. Linked with accurate measures of genome replication this provides a defined framework to analyse the developmental cycle and to investigate and provide new insights into the effects of antibiotic treatments. Removal of penicillin allows recovery of the normal developmental cycle by 10–20 hrs and the process occurs by budding from aberrant RBs.

## Introduction

*Chlamydia trachomatis* is a major human pathogen responsible for ocular and sexually transmitted diseases [1], [2]. It is an obligate intracellular bacterium which grows and divides within a cytoplasmic structure known as an ‘inclusion’ [3], [4]. The inclusion is a unique biological organelle adapted from host cell exocytic vesicles induced by the infecting bacterium [5], [6]. Host cell infection begins by the binding of the infectious form of the organism, the elementary body (EB), onto the host cell cytoplasmic membrane. Uptake is efficient and EBs differentiate to form reticulate bodies (RBs) which are the metabolically active, replicating form of the microorganism. Active expression of chlamydial genes is required for this process [7]. Once an active infection is established RBs divide by binary fission, within inclusions, before undergoing further differentiation and condensation of their DNA to form new EBs that are released from the host cell upon lysis.

The chlamydial developmental cycle described above takes from 30 to 72 hrs to complete. High quality video imaging technology now makes it possible to observe and record the biology of chlamydial infection in detail Technical difficulties of measuring infectivity present problems of standardisation because some strains of *C.trachomatis* have high particle to infectivity ratios and need to be centrifuged onto host cells to initiate infection [8]. To avoid this we have used the standard LGV strain L2 which has a particle to infectivity ratio of 1.0 and does not require centrifugation. Surprisingly, few studies have used DNA quantification as a standard measure of the developmental cycle despite the suggestion a decade ago [9]. Recently, we have described quantitative PCR assays which allow the precise measurement of chlamydial DNA for both the chromosome and plasmid, overcoming the problems of quantification of chlamydial replication [10]. The combination of chromosomal and plasmid quantification assays provides a controlled way of evaluating replication and we have proposed that the chlamydial developmental cycle should be considered not in terms of time but in terms of bacterial chromosomal divisions [11]. The *C.trachomatis* developmental cycle is typically completed within 8 bacterial chromosomal divisions, yielding some 500 infectious EBs per infected host cell. The developmental cycle has three distinct phases: early, when EBs become reorganised to RBs; mid/exponential, when cell division is at maximum and late, when replication slows and mature inclusions are forming with EBs. The minimum generation time that occurs during exponential growth is 2.6 to 4.6 hrs [11], [12].

When chlamydia are exposed to beta lactam antibiotics the developmental cycle slows and the transition to EB becomes retarded. This results in aberrant RB formation where the RBs become enlarged and the developmental cycle enters a persistent, non infectious state. The mechanism by which this occurs is not known, although penicillin typically acts by blocking peptidoglycan (PG) biosynthesis through binding with penicillin binding proteins [13], [14]. However, the presence of PG in the chlamydial cell envelope has yet to be established although the genes encoding the enzymes for PG biosynthesis are present [15], [16]. This is known as the chlamydial anomaly [17], [18] and has been more recently described as the chlamydial peptidoglycan anomaly [19], [20]. PG has not been seen to form a sacculus around EBs or RBs, thus no structural role has been determined. Recently, we performed studies on the effects of penicillin treatment on chlamydial replication during the exponential phase of the developmental cycle. These studies showed that the mechanism for the segregation of chlamydial DNA is not essential for its replication [11]. It is important to understand the process by which persistent infection can be induced by antibiotic treatment as this may be a reservoir for future spread of infections.

The absence of a simple means to manipulate the *C.trachomatis* genome also restricts research into many processes including PG biosynthesis. Observational studies suggest that penicillin treatment does not affect EB to RB differentiation, although it does inhibit chromosomal condensation and, consequently, the transition from replicating RB to infectious EB in the later stages of the developmental cycle [21]–[26]. Penicillin treated chlamydial cultures contain large, aberrant RBs with multiple copies of the chromosomal DNA [11]. EM and infectious recovery studies show that when penicillin is removed from the cultures infectious EBs can later be recovered, although this is dependent on the timing and dose [22]. EM analyses are subjective and therefore the origins of these EBs are unclear. It is possible that they arose in one of three ways: through new infection, from budding of aberrant RBs and by division/septation of aberrant RBs. The latter two mechanisms have been proposed in a 40 year - old study [22]. The aim of this study was to apply high resolution imaging and DNA assays to investigate the effects of penicillin treatment on chlamydial morphology and chromosomal replication early in the developmental cycle and evaluate the effect of penicillin removal. Live cell imaging of chlamydial inclusions make it possible to follow the effects of penicillin addition and removal on chlamydial cultures in real time. The application of the latest video imaging techniques and qPCR analyses allow us to re–evaluate the original descriptions of the morphological effects of penicillin on the chlamydial developmental cycle.

## Results and Discussion

### 1. The Effect of Penicillin on the Early Stage of the Chlamydial Developmental Cycle

We used penicillin G at 100 units/ml, a concentration that had previously been shown to be optimal for retarding the chlamydial developmental cycle when added at the mid log phase [11]. In this previous study application of penicillin G at the mid point of the developmental cycle, at the 4^th^ binary division when the chlamydia are entering exponential growth, has the immediate effect of stopping chlamydial cell division. However, the effects of adding penicillin G at the start of the developmental cycle have not been well characterised.

**Figure 1:**
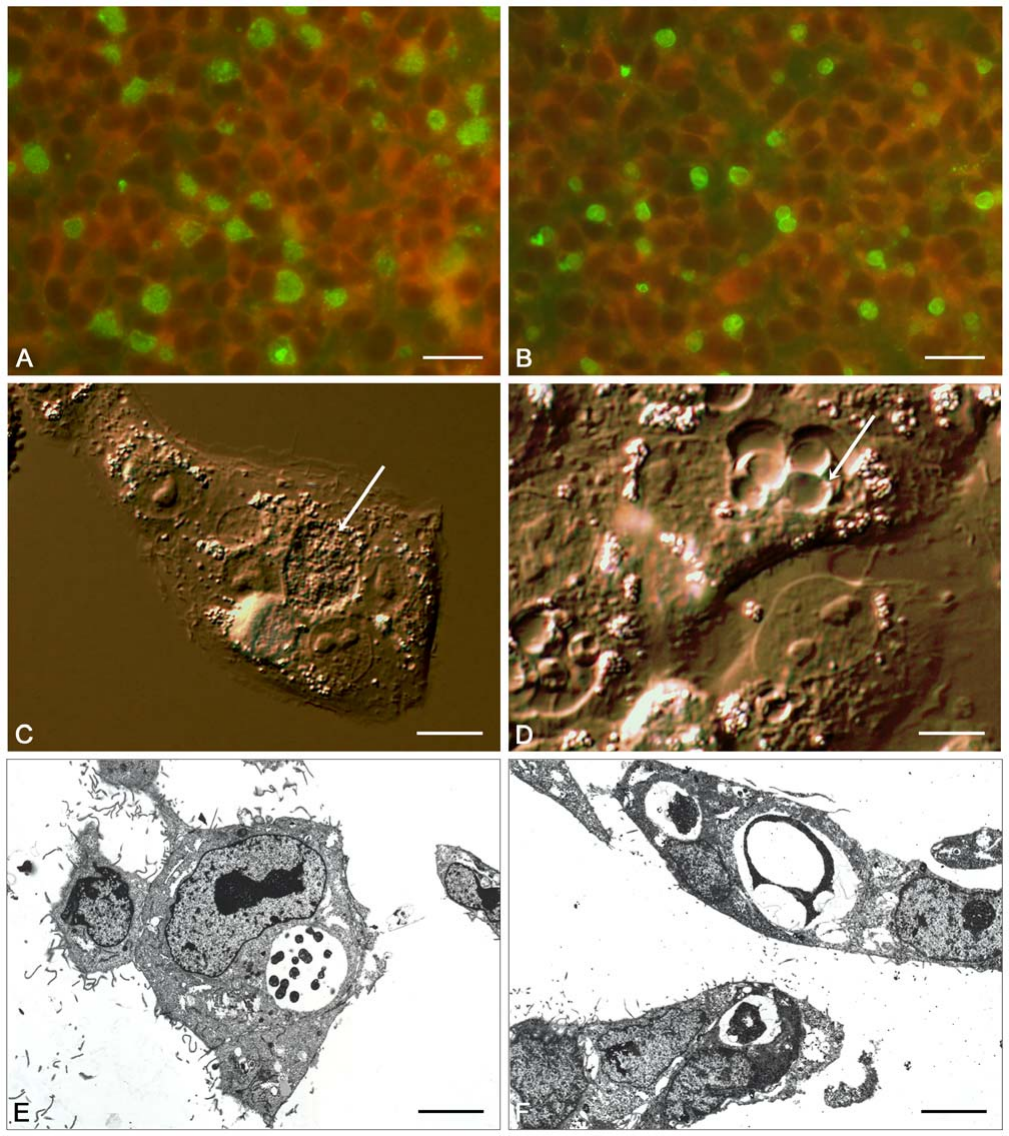
Comparison of *C.trachomatis* infected cells in the absence and presence of penicillin at 24 hrs post infection. Immunofluorescence staining of *C.trachomatis* inclusions in the absence (A) and presence (B) of penicillin. Monoclonal antibody 29 which binds to chlamydial LPS was visualised with FITC–conjugated anti-mouse IgG (green). Panels C (no penicillin) and D (with penicillin) show higher magnification interference contrast microscope images of inclusions, the white arrow highlights the different morphology of the inclusions. Panels E and F show transmission electronmicrographs of inclusions in the absence (E) and presence (F) of penicillin. The scale bar represents 24 µm for panels A and B and 4 µm for panels C–F.

TEM at 24 hrs p.i. showed that inclusions mainly carried a single large, aberrant reticulate body (Figure 1). However, TEM is a subjective procedure and does not allow large-scale scanning of fields. Therefore, to obtain an overall assessment of the effects of penicillin on chlamydial development, immunofluorescence (IF) on fixed, infected cells at lower magnifications, but the same time points, was also performed (Figure 1). Inclusions were relatively small compared to the host cells at 24 hrs p.i. and with fixed cells it was not possible to resolve individual RBs by IF at this time point. As an alternative approach we used interference contrast microscopy which showed that chlamydial inclusions developed in the presence of penicillin and that these appear as large vesicles rather than the ‘normal’ movement filled inclusions associated with the chlamydial developmental cycle; up to four RBs per inclusion could be observed (Figure 1).

Interference contrast microscopy of penicillin–treated, live, chlamydia–infected cells shows that, within a field, all EBs have developed into RBs and some have undergone a limited amount of division but then have failed to divide further and become enlarged instead. Counting of such RBs suggests that a maximum of four RBs can be detected per inclusion indicating that up to two bacterial divisions are possible under penicillin selection when the antibiotic is present from the start of the infection. Thus penicillin does not prevent EB to RB differentiation nor does it necessarily prevent the first or second bacterial division. However, RBs are unable to divide further whereas, by contrast, during the normal developmental cycle (untreated with penicillin) they have undergone four divisions by 24 hrs p.i. (i.e inclusions contain up to 16 dividing RBs).

### 2. The Prolonged Effect of Penicillin

**Figure 2:**
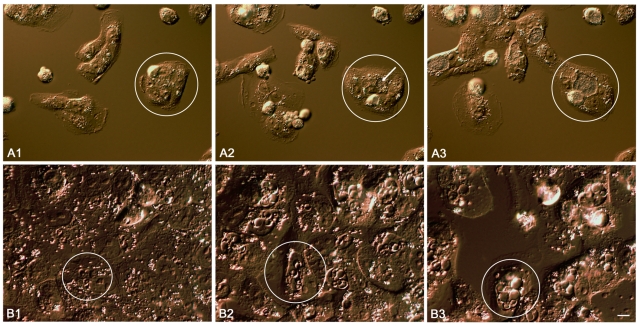
Interference contrast time lapse images of the *C.trachomatis* developmental cycle (A) and in the presence of penicillin (B). A cell carrying an inclusion is circled in successive frames for each set of panels A1 to A3 and B1 to B3 which individually represent an image taken at the 6, 18 and 30 hrs post infection. The location of a normal inclusion soon after its first appearance is arrowed in panel A2 (18 hrs p.i.). This figure should be viewed in conjunction with the supporting video S1 (panels A1 - 3) and S2 (panels B1 - 3). The scale bar represents 4 µm.

At 48 hrs p.i. the normal developmental cycle of *C.trachomatis* L2 in BGMK cells is almost complete and in late phase. By this time the chlamydia have undergone, on average, 7/8 bacterial cell divisions. Thus the inclusion can be considered as an intracellular microcolony entering into stationary phase and containing some 500–1,000 bacteria, most of which are present as EBs. The normal chlamydial developmental cycle from infection to cell lysis was observed by time lapse interference contrast microscopy (see Figure 2A and supporting video S1). The high resolution of this microscopy system permitted the visualisation of normal RBs and showed inclusions with active bacterial movement. This is not due to the presence of granular glycogen because inclusions from plasmid free *C.trachomatis* L2, which is unable to synthesise glycogen [27], show the same effect (data not shown). Fields were photographed at one minute intervals and these data have also been compiled as a time lapse video of the developmental cycle (supporting video S1).

**Figure 3:**
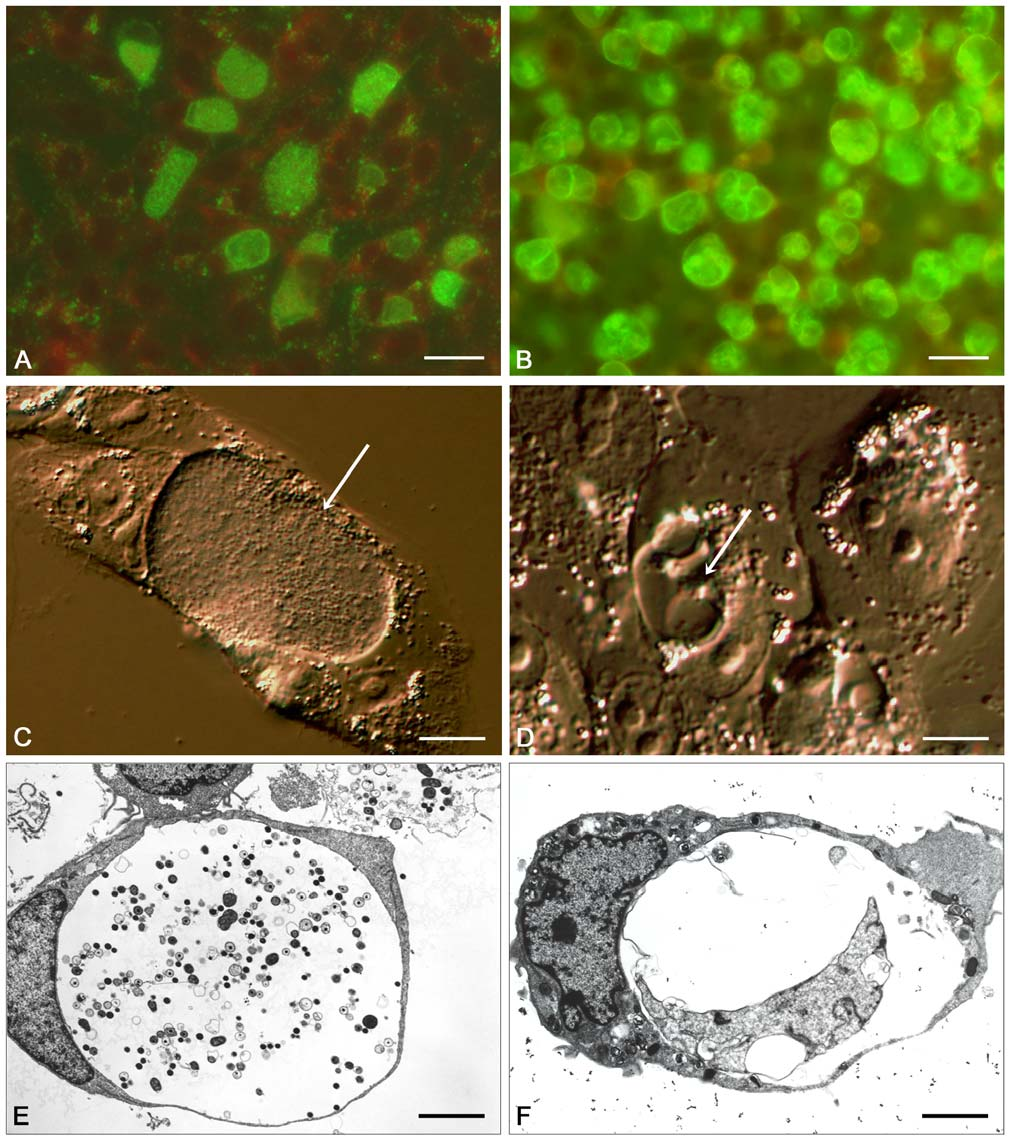
Comparison of *C.trachomatis* infected cells in the absence and presence of penicillin at 48 hrs post infection. Immunofluorescence staining of *C.trachomatis* inclusions in the absence (A) and presence (B) of penicillin. Monoclonal antibody 29 was visualised with FITC–conjugated anti-mouse IgG (green). Panels C (no penicillin) and D (with penicillin) show higher magnification interference contrast microscopy images of inclusions, the white arrow highlights the different morphology of the inclusions. Panels E and F show transmission electronmicrographs of inclusions in the absence (E) and presence (F) of penicillin. The presence of crescent–shaped aberrant RBs (in panel F) is not an artefact of fixation for EM as these are commonly seen to form in real time (see supporting video S2). The scale bar represents 24 µm for panels A and B and 4 µm for panels C–F.

By contrast, chlamydial inclusions in penicillin-treated cells continued to enlarge over the period from 24 to 48 hrs p.i. as did the RBs within them (Figure 2B and supporting video S2). TEM at 48 hrs p.i. showed the typical aberrant RBs which had enlarged from the 24 hr time point (Figure 3). The presence of up to 4 RBs per inclusion was also confirmed by IF (the larger aberrant RBs were easily resolvable) and these data also showed that no significant further bacterial cell division had occurred (Figure 3). The presence of multiple aberrant RBs within an inclusion was previously suggested to have arisen only by fusion of separate inclusions [22]. Whilst this is a possible mechanism it is clear from our studies that the aberrant RBs divide initially by binary fission and that this is the mechanism that accounts for the presence of up to 4 RBs per inclusion.

### 3. Removal of Penicillin

Previous reports on removal of Penicillin from the chlamydial culture medium suggest that the normal developmental cycle was recovered by budding of normal RBs from aberrant enlarged RBs, the nascent normal RBs then divide by binary fission, following the course of a normal developmental cycle [22]. This study used *C.psittaci* in L cells and samples were taken for TEM. We also removed penicillin from the culture medium at 20 hrs p.i. using *C.trachomatis*, however, for this chlamydial species the developmental cycle is slower. Microscopical observation of the culture revealed the emergence of the normal developmental cycle 10–20 hrs after removal of penicillin although this did not occur for all inclusions (supporting video S3). Our sampling by TEM was unable to capture the early moments when resumption of the developmental cycle occurred. We reasoned that whilst this event is quite common (most inclusions revert back to the normal lytic developmental cycle) it occurs across a broad time range, therefore capturing the precise moments associated with this change using fixed sections of chlamydia infected cells is problematic. This is especially the case with TEM where selection of images is subjective, thus a large set of supporting independent TEMs would be needed to draw meaningful conclusions. Given that each sample is fixed for the TEM process it is not possible to know how the infection would progress when assessing TEMs obtained from a single time point. Thus time lapse interference contrast photomicroscopy offers an alternative and more flexible approach for undertaking this kind of study as the cells are not fixed and hence it is possible to follow the developmental process sequentially. Incorporation of the time lapse sequence of images into a video stream (supporting video S3) allows the inclusions in which the lytic developmental cycle has resumed to be identified and then tracked back to the time point at which EBs start to form.

It is clear that the aberrant RBs do not revert back to smaller ‘normal’ RBs to resume the developmental cycle. In all of the inclusions we observed the large RBs remained present and relatively immobile within inclusions until cell death this is consistent with recent observations where penicillin was added at mid log phase in the developmental cycle [28]. When the normal development cycle was resumed it did not resolve by reductive division of the aberrant RBs and hence a single aberrant RB containing multiple chlamydial genomes did not revert back into normal RBs. The appearance of RBs within an existing inclusion also excluded the possibility that the inclusion arose by infection of the cell by a quiescent EB. The video shows appearance of normal RBs close to the large aberrant RBs; the normal RBs rapidly move to the inclusion membrane and then the inclusion fills as they divide. Thus, by elimination, our data support the proposal that a budding process, observed in a single TEM forty years ago, is the most likely mechanism by which the developmental cycle is resolved.

**Figure 4:**
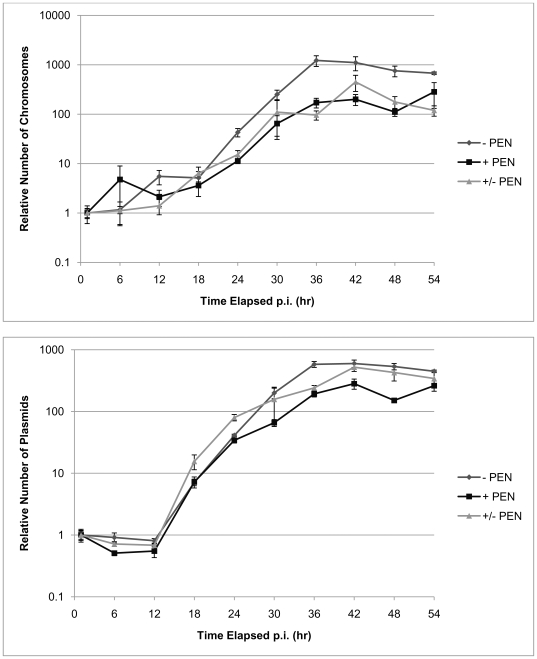
The effects of penicillin addition and removal on chromosomal and plasmid replication during the developmental cycle of *C.trachomatis* - infected BGMK cells. Cells were harvested for qPCR analysis at 1, 6, 12, 18, 24, 30, 36, 42, 48 and 54 hrs p.i. The relative number of chlamydial plasmids (A) and chromosomes (B) was determined using real time qPCR TaqMan assays.

qPCR analyses were also performed on samples of infected cells at different time points during the chlamydial developmental cycle in the presence or absence of penicillin treatment or when penicillin was removed. These data (Figure 4) show that chromosomal and plasmid DNA replication are unaffected by the addition and removal of penicillin with both markers amplifying by 500 fold and thus confirming that cytokinesis and chromosomal DNA replication are not linked when penicillin is added to the culture. The phenomenon of ‘persistence’, where the chlamydial developmental cycle becomes retarded, is also induced by a number of other factors including metabolite depletion and the action of gamma interferon [29], [30]. Application of the photomicrosopy protocols developed here together with DNA analyses will help in visualising and understanding the morphological processes that result from these treatments.

The data presented here clearly define the morphological effects of penicillin treatment on the chlamydial developmental cycle. The use of time lapse photomicroscopy linked with qPCR data on chlamydial chromosomal DNA and plasmid replication provides a reproducible framework within which to begin molecular analyses of the chlamydial developmental cycle. This approach will allow cross comparison of data between different chlamydial strains and host cells. Furthermore it will now be possible to begin a detailed molecular dissection of the chlamydial developmental cycle with respect to bacterial cell divisions and the morphological changes brought about by penicillin. It is hoped that a systematic dissection of the effects of penicillin treatments on expression of genes involved in PG biosynthesis can begin to unravel the chlamydial ‘anomaly’ and allow a better understanding of the phenomenon of chlamydial persistence.

### Conclusions

1. Penicillin treatment from the start of infection does not prevent attachment, uptake and the establishment of an early inclusion: EBs are taken up and differentiate into RBs.
2. RBs formed under penicillin selection (and kept under selection) are able to divide by binary fission for a maximum of two divisions.
3. Prolonged exposure to penicillin leads to enlargement of the RBs and subsequent expansion of the inclusion.
4. Chlamydial chromosomal and plasmid DNA replication is unaffected by Penicillin treatment from the start of infection.
5. Removal of penicillin allows resumption of the developmental cycle, aberrant enlarged RBs do not revert back to normal RBs but retain their larger size and structure and remain relatively immobile within the inclusion.
6. The normal developmental cycle is re established and becomes visible some 10–20 hrs after the removal of penicillin, however not all inclusions resume the developmental cycle when penicillin is removed.

## Materials and Methods

### *Chlamydia trachomatis*

*C. trachomatis* L2/434/Bu (ATCC - VR902B) was plaque purified and cultured in Buffalo green monkey kidney (BGMK) cells with cycloheximide (1 µg/ml). The cell cultures and chlamydial inocula were mycoplasma negative. BGMK cells were selected for chlamydia culture for this study because they are clonal and they grow and divide evenly. This allows clear visualisation of chlamydial inclusions by phase and interference contrast micrcoscopy. BGMK cells were grown in Dulbecco's Modified Eagle's Medium (DMEM) supplemented with foetal calf serum (FCS, 10% v/v). EBs were purified from Urografin gradients as previously described [31] and their infectivity was evaluated by serial titration and staining of inclusions using an in-house monoclonal antibody specific to chlamydial LPS (Mab29) [32].

### High Quality Digital Time Lapse Video Photomicroscopy

Digital time lapse images were captured by growing BGMK cells infected with *C.trachomatis* within a sterile, stainless steel chamber, which was completely sealed containing a well with 1.5 ml of medium and a volume of filtered air (35 ml) containing 5% CO_2_. Cells could be grown in this chamber for up to five days.

The chamber had a 55.5 mm internal diameter (63 mm outside diameter) and 21 mm inside height (26.5 mm external height). It unscrewed around its equator into two halves. Details of the chamber can be seen in supporting Figure S1. The bottom half contained a funnel shaped well 19 mm in diameter at the base and 9 mm deep which held the sample. The floor of the well was a glass coverslip (25 mm diameter no. 1 coverslip) allowing a 19 mm diameter viewing window on which the cells could grow and be observed using an inverted microscope. The top half of the chamber had a 30 mm window also sealed with a large 35 mm diameter no. 1 coverslip. Both windows were cleaned and sealed into the steel chamber using a proprietary aquarium silicone sealant which is free from any fungicide or anti mould agent. This arrangement allowed time lapse observation at all magnifications up to 100× in differential interference contrast or phase contrast. Each frame was taken at 1 minute intervals and run as a video at 25 frames/sec (see supporting video S1, S2, and S3).

The chamber had 12×1 mm holes drilled through the screw thread of the base. After placing cells in the centre well, the top was partially screwed down to leave the holes open and placed in a standard incubator to equilibrate in a 5% CO_2_/air mixture for four hours. After this the chamber was fully screwed down to cover the holes and further sealed by placing a broad elastic band around the equator of the chamber to cover the join. The inside surface of the elastic band was coated with silicone grease, minimizing any change in the gas composition within the chamber due to diffusion effects.

The chamber was placed within a heated box at 37°C fitted to an Olympus IX81 inverted research microscope and a series of images captured. Each photograph was 2,040×1,536 pixels in resolution. Autofocus was used to ensure that a sharp image was obtained during the long capture period. The sequences produced were between 6000 and 7500 frames long. The camera was an Olympus DP71 camera controlled using Olympus Cell P software.

### Transmission Electron Microscopy

At the specified time intervals, the cell monolayers infected with *C. trachomatis* L2/434/Bu (moi = 1.0) were viewed by phase contrast microscopy. Following washing with PBS, monolayers were fixed in 3% glutaraldehyde in 0.1% cacodylate buffer pH 7.4 and prepared for TEM as previously described [33].

### Immunofluorescence Staining of *C.trachomatis*

BGMK cells were grown on 13 mm coverslips in 24 well trays. The cells were infected with *C.trachomatis* L2 at a MOI of 1.0 in DMEM and 10% FCS or DMEM and 10% FCS with 100 units/ml Penicllin G. At 24 and 48 h post infection the cells were washed twice with ice cold PBS and then fixed with methanol at −20°C for 15 min. A monoclonal antibody that binds to chlamydial LPS (Mab29) was used to detect chlamydia and fluourescence detected as previously described [32].

### Time Course of Infection and Extraction of DNA for Real Time PCR Analyses

BGMK cells grown to confluence in 96 well trays were infected with purified *C. trachomatis* L2/434/Bu EBs at MOI = 1.0. EBs were allowed to adsorb to cells for 1 hr at 37°C; cells were then washed with PBS to remove any residual unabsorbed EBs. The infected cells were overlaid with 100 µl culture medium and incubated at 37°C in 5% CO_2_. For each time point cells were infected in triplicate, and the infection was stopped at 6, 12, 18, 24, 30, 36, 48 and 54 hrs post infection (p.i.). Samples were stored after snap freezing at −80°C. For penicillin - treated cultures, medium containing 100 units/ml penicillin G (Sigma, Poole, Dorset) was added at the time of infection. To assess the effects of penicillin removal on cell cultures that had been treated with penicillin from the time of infection, cells were washed three times with PBS and the cell culture medium was replaced with antibiotic free DMEM containing 5% FCS. Genomic and plasmid DNA was extracted in a microplate format following a well described protocol [10]. The residue was then resuspended in 100 µl nuclease-free water. Samples were diluted 1 in 100 prior to qPCR analysis.

### Real-Time QPCR

Our quantitative real-time PCR protocol to determine the absolute number of chlamydial plasmids and genomes in samples using 5′- exonuclease (TaqMan) assays with unlabelled primers and carboxyfluorescein/carboxytetramethylrhodamine (FAM/TAMRA) dual-labeled probes has been described previously [10], [11].

## Supporting Information

Figure S1 The re-usable chamber for time lapse interference microscopy. Panel A shows the top section of the chamber. Panel B shows the bottom section with the well for media and the replaceable coverslip for growing cells, the holes for gassing are arrowed. Panel C is the assembled chamber.
(0.75 MB TIF)

Video S1 Time lapse video stream showing the normal chlamydial developmental cycle in McCoy cells. A cluster of cells in which an inclusion develops is circled, an inclusion (arrowed) becomes visible by 12 hrs post infection (p.i.).
(16.4 MB MOV)

Video S2 Time lapse video stream showing the chlamydial developmental cycle with penicillin present from the start of the infection. An infected cell is circled and aberrant penicillin induced RBs start to appear by 6 hrs post infection (p.i.) and are clearly visible by 12 hrs p.i. Prior to lysis this cell contains 4 aberrant RBs.
(10 MB MOV)

Video S3 Time lapse video stream showing the effects of penicillin removal at 20 hrs post infection on the chlamydial developmental cycle. The digital recording starts at approximately 24 hrs p.i following equilibration of the chamber with CO2. The time points refer to hours post removal of penicillin and the circles highlight examples of inclusions containing penicillin - induced RBs. In circle A the appearance of normal RBs near to the single large aberrant RB is shown and occurs by 12 hrs, RBs then move to the edge of the inclusion. In circle B there is a cluster of 4 aberrant RBs, the appearance and motion of normal RBs becomes clearly visible by 24 hrs.
(15.2 MB MOV)
