# Supplementary figures and images for: Penicillin Induced Persistence in Chlamydia trachomatis: High Quality Time Lapse Video Analysis of the Developmental Cycle
Source: PLoS One. 2009 Nov 6;4(11):e7723. doi: 10.1371/journal.pone.0007723 (PMC2769264; doi:10.1371/journal.pone.0007723)

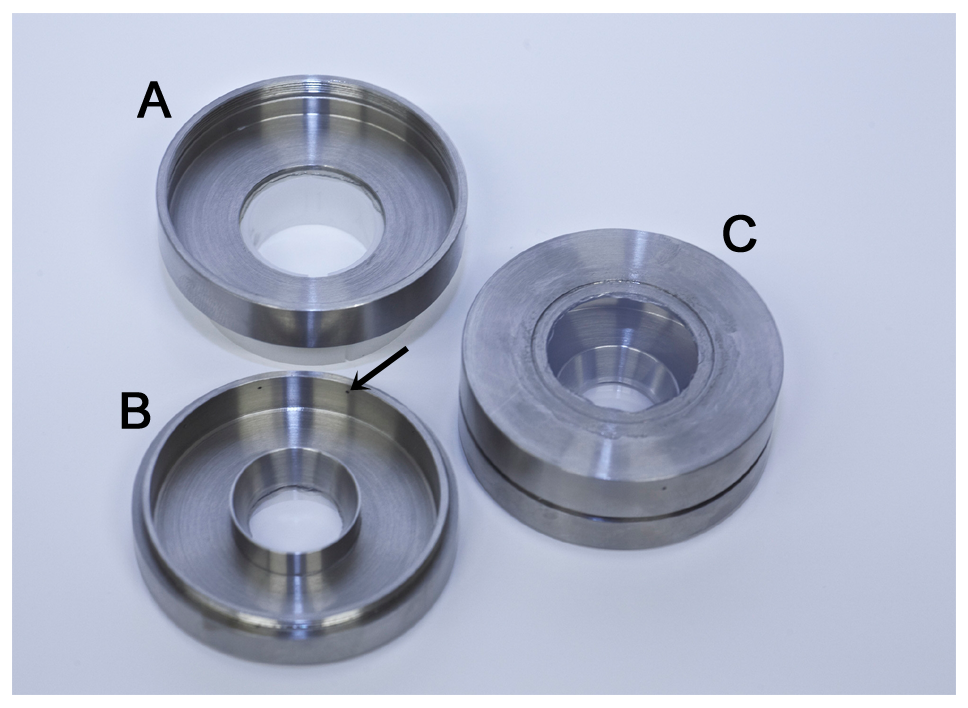

Supplement: Figure S1 — The re-usable chamber for time lapse interference microscopy. Panel A shows the top section of the chamber. Panel B shows the bottom section with the well for media and the replaceable coverslip for growing cells, the holes for gassing are arrowed. Panel C is the assembled chamber. (0.75 MB TIF) [file pone.0007723.s001.tif]
